# Tail autotomy works as a pre‐capture defense by deflecting attacks

**DOI:** 10.1002/ece3.7213

**Published:** 2021-03-04

**Authors:** Laura A. Naidenov, William L. Allen

**Affiliations:** ^1^ Department of Biosciences Swansea University Swansea UK

**Keywords:** animal coloration, antipredator defense, autotomy, caudal autotomy, deflection, squamate

## Abstract

Caudal autotomy is a dramatic antipredator adaptation where prey shed their tail in order to escape capture by a predator. The mechanism underlying the effectiveness of caudal autotomy as a pre‐capture defense has not been thoroughly investigated. We tested two nonexclusive hypotheses, that caudal autotomy works by providing the predator with a “consolation prize” that makes it break off the hunt to consume the shed tail, and the deflection hypothesis, where the autotomy event directs predator attacks to the autotomized tail enabling prey escape. Our experiment utilized domestic dogs *Canis familiaris* as model predator engaged to chase a snake‐like stimulus with a detachable tail. The tail was manipulated to vary in length (long versus short) and conspicuousness (green versus blue), with the prediction that dog attacks on the tail should increase with length under the consolation‐prize hypothesis and conspicuous color under the deflection hypothesis. The tail was attacked on 35% of trials, supporting the potential for pre‐capture autotomy to offer antipredator benefits. Dogs were attracted to the tail when it was conspicuously colored, but not when it was longer. This supports the idea that deflection of predator attacks through visual effects is the prime antipredator mechanism underlying the effectiveness of caudal autotomy as opposed to provision of a consolation prize meal.

## Graphical abstract

In an experiment on domestic dogs chasing model prey, we found that attacks were directed to an autotomized body parts when it was conspicuously colored but not when it was longer. This supports the hypothesis that autotomy is principally effective because of perceptual deflection of attacks rather than because it offers predators a “consolation prize” meal.

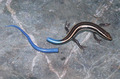

## 1 INTRODUCTION

**Figure 1:**
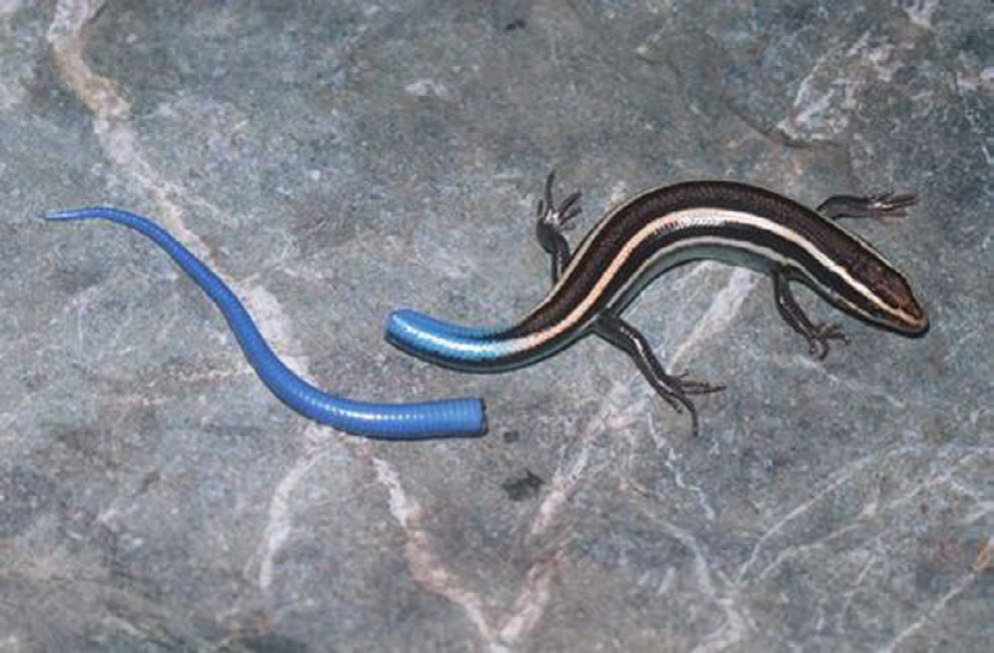
Western Skink (*Plestiodon skiltonianus*) tail detached from body following caudal autotomy (©2005 William Leonard, used with permission)

Autotomy, the ability of an animal to shed a body part without any external force, is an extreme and dramatic antipredator adaptation (Emberts et al., 2019; Maginnis, 2006). Autotomy is widespread among animals, having independently evolved at least nine times, and is common among insects, crustaceans, amphibians, and reptiles (Emberts et al., 2019). It is most frequently studied in lizards where tail shedding (“caudal autotomy”; Figure 1) in response to threat is present in 15/18 lizard families (Bateman & Fleming, 2009).

The effectiveness of caudal autotomy in reducing predation is well established (reviewed in Arnold, 1984; Bateman & Fleming, 2009; Emberts et al., 2019) but few studies have investigated the underlying antipredator mechanism(s). Autotomy that occurs after a tail has been grasped by a predator likely works because the predator is occupied with subduing the tail, and the time required to drop the tail and resume pursuit of the body gives prey an opportunity to escape. However, autotomy is also frequently performed by prey pre‐capture or when a tail has only been lightly touched (Arnold, 1984). In this scenario, there are two principal nonexclusive ways prey might benefit from autotomy (Arnold, 1984). The deflection hypothesis proposes that autotomy has the effect of directing the predator's point of attack toward the tail, enabling the prey's body to escape (Cott, 1940; Edmunds, 1974; Ruxton et al., 2018). The sudden loss of a body part may be a stimulating sensory event for the predator, drawing the attack to the tail via perceptual exploitation (Humphreys & Ruxton, 2018). Alternatively or additionally, autotomy might work simply by providing a “consolation prize” to predators that are about to catch the prey animal, so that the predator breaks of pursuit of the body to consume a small but certain meal, enabling the prey to escape while the predator consumes the consolation. Consuming the tail might be optimal for the predator because it does not spend additional energy on what might be an unsuccessful hunt (Kacelnik & Bateson, 1996), and immediate consumption avoids the risk of losing the autotomized tail to another foraging animal. Here, we test these two nonexclusive mechanistic hypotheses for the antipredator benefit of autotomy.

Current support for the deflection hypothesis for pre‐capture autotomy comes from evidence that traits associated with caudal autotomy such as conspicuous tails have a deflective effect. In a laboratory‐based predation experiment, Cooper and Vitt (1985) found that attacks by scarlet kingsnakes (*Lampropeltis elapsoides*) were directed toward the tails of two species of juvenile skink (*Plestiodon fasciatus* and *P. laticeps*) in 50% of trials when the tail was unmanipulated conspicuous blue color, compared to just 7% of trials when tails were painted black to match body color. Biting the tail always led to autotomy and prey escape, whereas biting the body always resulted in prey capture and consumption. Several studies have also found that free‐ranging predator attacks on static model lizards are directed toward tails when they are conspicuously colored compared to cryptic controls (Bateman et al., 2014; Castilla et al., 1999; Fresnillo et al., 2015; Heninger et al., 2020; Watson et al., 2012). Longitudinal body stripes have been proposed to contribute to deflection through “dazzle” mechanisms that lead predators to underestimate the escape speed of the lizard and therefore direct attacks caudally (Murali & Kodandaramaiah, 2016, 2018). Color and pattern‐based deflection has also been supported in other taxonomic groups (Fish: Kjernsmo & Merilaita, 2013; Mammals: Powell, 1982; Amphibians: Van Buskirk et al., 2004). Additionally, motion has also been shown to help deflect attacks on lizards through both preautotomy tail waving (Telemeco et al., 2011) and postautotomy tail thrashing (Dial & Fitzpatrick, 1983; Higham et al., 2013). In phylogenetic analyses, lizard autotomy was found to precede the evolution of conspicuous tails and then become tightly positively linked, with lineages that lose autotomy ability generally also losing conspicuous tails (Murali et al., 2018). This makes sense as diverting an attack to the tail is unlikely to prevent capture unless the tail can be shed. Overall, this points to a multicomponent “autotomy phenotype” based on deflection of attacks (Emberts et al., 2019), that is an effective trait combination despite the increased conspicuousness of colorful moving tails and physiological and energetic costs of tail loss (Bateman & Fleming, 2009; Cooper & Vitt, 1985). However, to date experiments have either used static models without the ability to shed their tail (Bateman et al., 2014; Castilla et al., 1999; Fresnillo et al., 2015; Heninger et al., 2020; Watson et al., 2012), or investigated autotomy occurring after predators had already captured the lizard's tail in laboratory setting (Cooper & Vitt, 1985), so there is no direct evidence that pre‐capture autotomy is effective because of deflection. Results are consistent with pre‐capture autotomy working because it offers predators a consolation prize, with tail color and movement separately deflecting predator attacks toward the consolation prize tail.

Support for the consolation prize hypothesis comes from the suggestion that slower‐moving lizards in open environments that might find escape difficult tend to be limited to basal tail breakages in order to provide a sufficiently rewarding consolation prize to predators, whereas faster lizards or those with easy access to cover usually autotomize just behind the point of capture to facilitate the “economy of autotomy” (Arnold, 1984; Cromie & Chapple, 2013). In a captive predation experiment, Daniels et al. (1986) found that autotomy was a more effective defense for adult compared to juvenile marbled geckos *Christinus marmoratus*, perhaps because adults had larger tails in both relative and absolute terms. Although this result is predicted by the consolation prize hypothesis, adults, and juveniles also differ in other potentially important ways such as escape speed and endurance. In King's skink *Egernia kingii*, it is instead juveniles that have relatively larger tails compared to adults and make greater use of autotomy, possibly because adults are able to defend themselves against smaller predators without recourse to autotomy (Barr et al., 2019). Across lizards, larger, diurnal, and more gracile species have relatively longer tails, a relationship consistent with tail investment as an adaptation to offer predators a relatively larger consolation prize (Fleming et al., 2013). Many other relationships between autotomy traits and life stage, ecotype, sex, behavior, and body size have also been identified (Hawlena, 2006; Ortega et al., 2014; Telemeco et al., 2011). However, while this body of evidence demonstrates that autotomy is tightly linked to costs for the prey, it is not yet clear how important the energetic reward of an autotomized tail is to predators (Emberts et al., 2019; Humphreys & Ruxton, 2018), preventing evaluation of the consolation‐prize hypothesis for caudal autotomy in any context, including pre‐capture autotomy.

In this experiment, we ask domestic dogs *Canis familiaris* to chase model “snakes” with tails that detach at a semirandom point in the chase prior to capture. We predict that if pre‐capture autotomy is an effective defense, attacks will sometimes be directed toward the automatized tail. By varying the length and color of the tails, we test the consolation prize and deflection hypotheses for the antipredator mechanism underlying pre‐capture autotomy. A finding that longer tails increase the proportion of tail attacks would support the consolation‐prize hypothesis as predators would be expected to attack the tail when it is larger and presumably more energetically rewarding. If conspicuous color increases the proportion of tail attacks, this would support the deflection hypothesis for pre‐capture autotomy, as deflection is more effective when stimuli are conspicuous (Humphreys & Ruxton, 2018). A positive interaction would suggest both mechanisms play a role in the effectiveness of pre‐capture caudal autotomy as an antipredator trait.

## 2 METHODS

### 2.1 Stimulus design

The “snake” stimuli were built using 36 mm diameter natural sisal rope, weighing 1.2 kg per meter, covered in fabric. Each snake comprised two sections, a body measuring 30 cm in length in all conditions and a tail measuring 10 cm in the short condition and 20 cm in the long condition. While this design increased the overall length of the snake in the long condition, it enabled the influence of tail length or appearance to be isolated from any effects of body length or appearance. Long and short tails were covered in either a conspicuous blue or inconspicuous green colored fabric in a 2 × 2 experimental design.

The fabrics colors selected were based on the dichromatic color vision of dogs who, like most mammals, lack medium wavelength cone photoreceptors (Neitz et al., 1989) and the color of the grass experimental background. Swatches of five different green and five different blue cotton fabrics were photographed alongside a Macbeth ColorChecker Passport color standard against a grass background using a calibrated Canon T2i camera. The images were analyzed in dog color space (Neitz et al., 1989) using the MicaToolbox plugin (Troscianko & Stevens, 2015) for ImageJ (Schneider et al., 2012). We calculated chromatic and achromatic just‐noticeable differences (JNDs) between each fabric swatch and a 5 cm^2^ patch of grass using MicaToolbox functions based on the Vorobyev‐Osorio receptor‐noise limited model (Siddiqi, 2004; Vorobyev & Osorio, 1998). We used an appropriate Weber fraction of 0.05 and cone ratios of 1:9 (SWS:LWS) (Mowat et al., 2008). We selected the inconspicuous green fabric that had the lowest sum of chromatic (1.78) and achromatic (2.26) JNDs and the conspicuous blue fabric that had the highest chromatic (17.65) but lowest achromatic (2.87) JNDs. Thus, the two fabrics differed greatly in chromatic contrast but not achromatic contrast.

**Figure 2:**
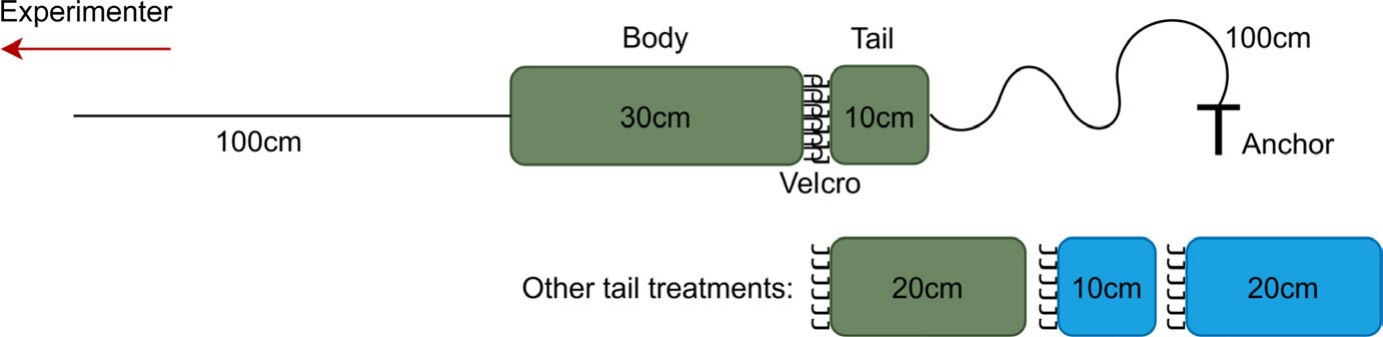
Diagram of the experimental setup

The tail and body parts were attached together by 4 cm^2^ Velcro sewn to the fabric. The body portion was attached to a 1 m long string at the “head” so that it could be pulled along the ground by the experimenter. The tail portion was attached to a 1 m long string anchored to the ground using a tent peg (Figure 2). This setup allowed the tail to detach from the body at the point in the chase when both strings went taut, simulating an autotomy event.

### 2.2 Procedure

The experiment took place in two urban parks popular with dog walkers (Singleton and Brynmill Park, Swansea, UK) between 9 a.m. and 5 p.m. on 8 days between 25 September and 21 October 2019. Dog owners were informed about the experimental objectives, the procedure, their right to withdraw at any point, and then made full written consent for their dog to participate. The procedure started with the experimenter getting the dog interested in the stimulus by playing with the dog, waving the snake and letting them see and smell it, sometimes with help or input from the owner(s). When the dog was engaged with the stimulus, for example by maintaining eye contact with it and attempting to grasp it, the experimental trials began. The dog owner maneuvered the dog to approximately 3 m away from the tail detachment point. At this point, the experimenter started moving the snake to get the dog's attention and initiate a chase. When the dog began chasing the snake the experimenter moved the snake away from the dog in a nonlinear path slightly slower than the dog's speed, aiming for the tail to detach when the dog was approximately 1 m away from the snake. After the body and tail separated, the experimenter continued to move the body away from the dog at the same speed until either the tail or body was touched by the dog. The experimenter recorded whether the dog continued to chase and attack the body, attacked the tail, or lost interest in the chase. If a dog lost interest in the chase, the experiment was halted. Trials where the dog lost interest were not included in analyses. A trial would also have been excluded if the dog caught the stimulus before the tail detached but this did not occur. If the dog continued to engage in the experiment, the experimenter changed the tail to the next treatment and the next trial began. Treatment order was counterbalanced using a balanced Latin square (ADBC, BCAD, CADB, DBCA; A = long green, B = short green, C = long blue, D = short blue).

Thirty‐four dogs participated in the experiment. Each dog participated in a maximum of four trials, one for each experimental condition. 61.8% (*n* = 21) of participants completed all 4 trials, 5.9% (*n* = 2) completed 3 trials, 14.7% (*n* = 5) completed 2 trials, and 17.6% (*n* = 6) completed 1 trial. In total, 106 trials were completed.

### 2.3 Analysis

The binary response variable, whether the dog attacked the body or tail, was modeled using a generalized linear mixed model with binomial link function, implemented using the R package *lme4* (Bates et al., 2015; R Core Team, 2018). The model included tail length (short versus long), tail color (green versus blue), and trial order as categorical fixed effects, and number of trials completed (1–4) as a continuous fixed effect. The interaction between tail color and length was also included. Individual dog identity was incorporated as a categorical random effect. The model was checked using the DHARMa package function testResiduals (Hartig, 2020) which identified no issues with the distribution of residuals, outliers, or dispersion of data.

## 3 RESULTS

**Figure 3:**
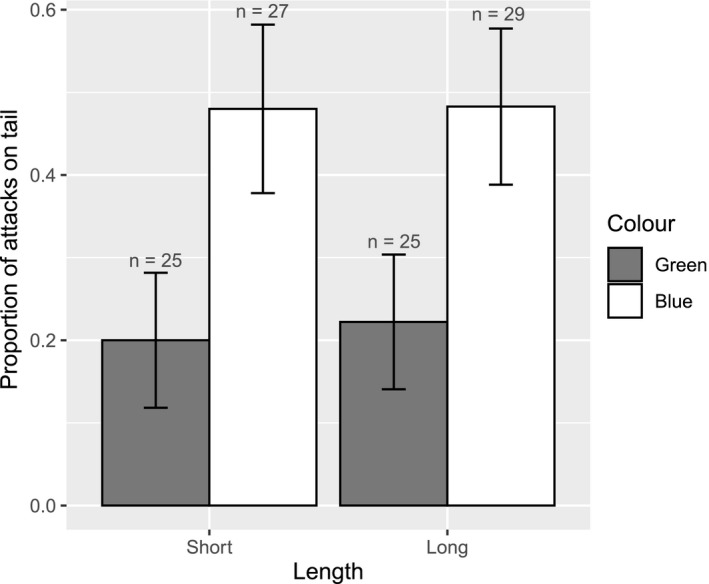
Proportion of attacks on the tail depending on the color and length of the tail. Error bars denote the standard error. Numbers above bars indicate the sample number

Overall, the tail was attacked on 35% of trials, and the body attacked on 65% of trials, indicating that pre‐capture caudal autotomy is effective as an antipredator defense. When the tail was blue, 70.3% of attacks were directed to the tail, while only 29.7% went to the tail when it was green. This difference was significant (*β* = 1.983, std = 0.823, *z* = 2.408, *p* = .016; Figure 3) and predicted by the deflection hypothesis for pre‐capture autotomy. There was no effect of tail length on attack location (*β* = 0.394, std = 0.830, *z* = 0.474, *p* = .635), nor was there a significant interaction between color and length (*β* = −0.159, std = 1.055, *z* = 0.150, *p* = .881).

There was no effect of trial number (*β* = −0.018, std = 0.261, *z* = 0.069, *p* = .945), the number of trials completed (*β* = −0.159, std = 0.358, *z* = 0.426, *p* = .670), or treatment order on attack location (DBCA versus BCAD *β* = 1.264, std = 0.824, *z* = 1.534, *p* = .125; DBCA versus CADB *β* = −2.215, std = 1.177, *z* = 1.883, *p* = .060; DBCA versus ADBC *β* = −0.178, std = 0.867, *z* = 0.206, *p* = .837).

## 4 DISCUSSION

In our experiment, tail loss on the model snake was an effective antipredator adaptation as attacks were directed toward tails on 35% of trials. This extends evidence that caudal autotomy is an effective antipredator adaptation to scenarios where autotomy is performed pre‐capture.

Further, our study suggests that the mechanism underlying the effectiveness of pre‐capture autotomy is deflection of predator attacks. Attacks on the snake's tail were much more frequent when the tail was conspicuous (70.3%) than inconspicuous (29.7%). This finding that autotomy is sometimes effective when tails are inconspicuous, but greatly enhanced when tails are conspicuous, leads us to conclude that the tail detachment event itself is likely to have a perceptual effect on predators that results in attacks being deflected toward the tail. The simplest explanation for this is that the novel movement associated with the autotomy event in conjunction with the conspicuous color directs predators to the tail by exploiting predator's perceptual biases for responding to salient novel cues (Humphreys & Ruxton, 2018; Schaefer & Ruxton, 2009). This conclusion is consistent with the comparative evidence that autotomy works via deflection, for example, the association between blue tails and autotomy and, in species with inconspicuous tails, other deflective traits such as tail wagging (Murali et al., 2018). The deflective effect of autotomy on the dogs’ responses might have been additionally enhanced by the noise produced by the Velcro coming apart at the separation point, but this remains an untested possibility—while acoustic deflection has been identified in several taxa (Humphreys & Ruxton, 2018) we are not aware of any studies linking autotomy with acoustic signals or cues.

As well as suggesting that the autotomy event is deflective, our findings extend previous evidence that conspicuous squamate tails have an antipredator function in deflecting attacks to less vital body regions. Previous studies have shown that conspicuous tails deflect attacks on static models (Bateman et al., 2014; Castilla et al., 1999; Fresnillo et al., 2015; Heninger et al., 2020; Watson et al., 2012) or before both capture and autotomy have taken place (Cooper & Vitt, 1985). However, our experiment shows that conspicuous tails are also effective in manipulating attack location after autotomy, but before the prey is caught, indicating another predation sequence under which conspicuous tails and autotomy are effective antipredator traits.

Finally, while prior experiments demonstrated the efficacy of blue tails as a deflective trait in avian and nonavian reptile predators, our result suggests that blue lizard tails are also likely to be an effective deflective trait against mammalian predators (Bateman et al., 2014; Castilla et al., 1999; Cooper & Vitt, 1985; Fresnillo et al., 2015; Heninger et al., 2020; Watson et al., 2012) adding to the known effectiveness of postautotomy tail thrashing against mammalian predators (Dial & Fitzpatrick, 1983).

We found no evidence of a tail length effect on attack location, or an interaction between tail length and color, so our experiment does not support the hypothesis that pre‐capture autotomy is effective because it offers predators a “consolation prize” of a smaller meal without risks associated with continued pursuit (Arnold, 1984; Humphreys & Ruxton, 2018). Full tail autotomy is rare in the wild (Pérez‐Mellado et al., 1997) and lizards modify the amount of tail shed depending on the circumstances to facilitate economic autotomy, generally 1–3 vertebrae above the point of capture in species with intravertebral autotomy (Arnold, 1984; Cromie & Chapple, 2013). However, whether tails are shed above the point of capture more than is necessary to detach from the predator in order to offer a larger consolation that is more likely to be accepted has not yet been investigated (Doughty et al., 2003; Humphreys & Ruxton, 2018; Naya et al., 2007). Observational studies and comparative analysis of fracture plane number and position would be valuable contributions to our understanding of autotomy (Emberts et al., 2019).

An alternative explanation for the lack of size effect is that the predation task did not replicate key aspects of natural predation on species with caudal autotomy. For experimental control and ethical reasons (Humphreys & Ruxton, 2018), our experiment made several concessions to simulating natural predator‐prey scenarios that perhaps had a greater impact on the strength of the test of the consolation‐prize hypothesis than the deflection hypothesis. We did not control, record, or manipulate subjects’ level of hunger, and while dogs are typically fed after exercise, satiation may have contributed to the lack of support for the consolation‐prize hypothesis. Satiation levels are known to affect foraging decisions, including the decision to accept a small but certain reward (tail) or gamble on a larger risky reward (body). The “budget rule” (Caraco et al., 1980; Stephens, 1981) that animals are risk prone when hungry and risk averse when satiated would predict a larger consolation‐prize effect in hungrier predators because they are less likely to accept the small tail. However, empirical evidence for the budget rule is patchy, as it is for other risky foraging “rules” such as acceptance of risk increasing when the absolute size of the reward increases (Kacelnik & El Mouden, 2013). Thus, the role hunger levels may have had in results is unclear but it is possible that controlling or manipulating satiation levels would identify contexts where the size of the consolation prize is important to predators.

Additionally, our stimuli did not offer any olfactory cues or independent postautotomy tail movement and had only abstract visual and tactile resemblance to snake prey (which the dogs tested are very unlikely to have been familiar with). This may have contributed to the lack of a size effect by reducing the likelihood the stimuli were perceived as potential prey. However, evidence suggests that use of domestic dogs as predators in a “chase” game with nonedible toys is likely to replicate predator behavior in an ecologically relevant way (Burghardt et al., 2016). Dogs’ play has been characterized as the expression of “natural” behaviors such as predation, but with less intensity and where the end goal is not reached, as in this experiment where the toy “snake” is not killed and consumed (Bradshaw et al., 2015). The “chase” task is similar to the hunting strategies of wolves *Canis lupus* and other Canidae that tend to hunt via pursuit, either individually or in groups, that are important predators of squamate reptiles (Díaz‐Ruiz et al., 2013). Nevertheless, it would be informative to conduct further investigations using energetically rewarding stimuli and with wild predators. Bateman and Fleming (2011) found the frequency of tail regeneration in brown anoles is higher in locations where pet cats are abundant compared to locations with feral cats or no cats, suggesting that escape from inefficient “playing” predators is more likely than from efficient foraging predators. How play versus real predation might influence the antipredator mechanism of caudal autotomy is unknown but it would be instructive to investigate whether breed information and behavioral upbringing influence responses to autotomy. Breeds selected for roles such as coursing or individuals that have been trained on hunting‐type tasks may display task behavior more similar to wild predators and be more likely to show a consolation‐prize effect.

None of the dogs tested likely had experience of reptiles with conspicuous tails as these species are not present in the UK. This further indicates that the tail color effect is a consequence of perceptual processes rather than a learned preference for prey with a particular color.

The presence of humans in the vicinity of the dog, in particular, the experimenter dragging the stimulus, likely influenced our results. It would be informative to replicate the experiment in a human‐free scenario. We predict that attacks on the tail would increase in the absence of humans because dogs are inclined to play with humans (Bradshaw et al., 2015), and the experimenter was dragging the body section.

Overall evidence suggests caudal autotomy works by deflecting attacks from the main body to the detachable tail, whether that be through sensory features of the autotomy event itself, bright coloration, some other conspicuous behavior, or a combination of stimuli (Higham & Russell, 2010; Humphreys & Ruxton, 2018; Pafilis et al., 2009). Our findings give insight into why squamates sometimes perform autotomy before capture in anticipation of attack (Arnold, 1984). That the autotomy event itself likely has a deflective effect, and that conspicuousness‐based deflection remains effective after autotomy, shows that prey has the option of gaining protection from predation by shedding tails before capture. This might have advantages over postcapture autotomy, for example reducing risks associated with allowing predators to come in very close proximity, which puts vital body parts at risk, or allows the chase on the body to resume before the prey has had enough time to make escape. We think that it would be interesting for future work to investigate in natural or laboratory populations the circumstances in which pre‐capture autotomy occurs, and the costs and benefits of this behavior.

## CONFLICT OF INTEREST

No conflicts of interest to declare.

## AUTHOR CONTRIBUTION

**Laura Naidenov:** Conceptualization (supporting); Formal analysis (equal); Methodology (equal); Resources (equal); Visualization (lead); Writing‐original draft (equal); Writing‐review & editing (equal). **William L. Allen:** Conceptualization (lead); Formal analysis (equal); Methodology (equal); Project administration (lead); Resources (equal); Supervision (lead); Visualization (supporting); Writing‐original draft (equal); Writing‐review & editing (equal).

## Data Availability

Data associated with this experiment can be downloaded at Dryad https://doi.org/10.5061/dryad.tdz08kpzj.

## References

[ece37213-bib-0001] Arnold, E. N. (1984). Evolutionary aspects of tail shedding in lizards and their relatives. Journal of Natural History, 18(1), 127–169. 10.1080/00222938400770131

[ece37213-bib-0002] Barr, J. I. , Somaweera, R. , Godfrey, S. S. , & Bateman, P. W. (2019). Increased tail length in the King’s skink, Egernia kingii (Reptilia: Scincidae): An anti‐predation tactic for juveniles? Biological Journal of the Linnean Society, 126(2), 268–275. 10.1093/biolinnean/bly196

[ece37213-bib-0003] Bateman, P. W. , & Fleming, P. A. (2009). To cut a long tail short: A review of lizard caudal autotomy studies carried out over the last 20 years. Journal of Zoology, 277(1), 1–14. 10.1111/j.1469-7998.2008.00484.x

[ece37213-bib-0004] Bateman, P. W. , & Fleming, P. A. (2011). Frequency of tail loss reflects variation in predation levels, predator efficiency, and the behaviour of three populations of brown anoles. Biological Journal of the Linnean Society, 103(3), 648–656. 10.1111/j.1095-8312.2011.01646.x

[ece37213-bib-0005] Bateman, P. W. , Fleming, P. A. , & Rolek, B. (2014). Bite me: Blue tails as a “risky‐decoy” defense tactic for lizards. Current Zoology, 60(3), 333–337. 10.1093/czoolo/60.3.333

[ece37213-bib-0006] Bates, D. , Mächler, M. , Bolker, B. , & Walker, S. (2015). Fitting linear mixed‐effects models using lme4. Journal of Statistical Software, 67(1), 1–48 10.18637/jss.v067.i01

[ece37213-bib-0007] Bradshaw, J. W. S. , Pullen, A. J. , & Rooney, N. J. (2015). Why do adult dogs ‘play’? Behavioural Processes, 110, 82–87. 10.1016/j.beproc.2014.09.023 25251020

[ece37213-bib-0008] Burghardt, G. M. , Albright, J. D. , & Davis, K. M. (2016). Motivation, development and object play: Comparative perspectives with lessons from dogs. Behaviour, 153(6–7), 767–793. 10.1163/1568539X-00003378

[ece37213-bib-0009] Caraco, T. , Martindale, S. , & Whittam, T. S. (1980). An empirical demonstration of risk‐sensitive foraging preferences. Animal Behaviour, 28(3), 820–830. 10.1016/S0003-3472(80)80142-4

[ece37213-bib-0010] Castilla, A. M. , Gosá, A. , Galán, P. , & Pérez‐Mellado, V. (1999). Green tails in lizards of the genus Podarcis: Do they influence the intensity of predation? Herpetologica, 55, 530–537.

[ece37213-bib-0011] Cooper, W. E. , & Vitt, L. J. (1985). Blue tails and autotomy: Enhancement of predation avoidance in juvenile skinks. Zeitschrift Für Tierpsychologie, 70(4), 265–276. 10.1111/j.1439-0310.1985.tb00518.x

[ece37213-bib-0012] Cott, H. B. (1940). Adaptive Coloration in Animals. Oxford University Press.

[ece37213-bib-0013] Cromie, G. L. , & Chapple, D. G. (2013). Is partial tail loss the key to a complete understanding of caudal autotomy? Austral Ecology, 38(4), 452–455. 10.1111/j.1442-9993.2012.02429.x

[ece37213-bib-0014] Daniels, C. B. , Flaherty, S. P. , & Simbotwe, M. P. (1986). Tail size and effectiveness of autotomy in a lizard. Journal of Herpetology, 20(1), 93–96. 10.2307/1564134

[ece37213-bib-0015] Dial, B. E. , & Fitzpatrick, L. C. (1983). Lizard tail autotomy: Function and energetics of postautotomy tail movement in *Scincella lateralis* . Science, 219(4583), 391–393. 10.1126/science.219.4583.391 17815319

[ece37213-bib-0016] Díaz‐Ruiz, F. , Delibes‐Mateos, M. , García‐Moreno, J. L. , María López‐Martín, J. , Ferreira, C. , & Ferreras, P. (2013). Biogeographical patterns in the diet of an opportunistic predator: The red fox *Vulpes vulpes* in the Iberian Peninsula. Mammal Review, 43(1), 59–70. 10.1111/j.1365-2907.2011.00206.x

[ece37213-bib-0017] Doughty, P. , Shine, R. , & Lee, M. S. Y. (2003). Energetic costs of tail loss in a montane scincid lizard. Comparative Biochemistry and Physiology ‐ A Molecular and Integrative Physiology, 135(2), 215–219. 10.1016/S1095-6433(03)00087-4 12781822

[ece37213-bib-0018] Edmunds, M. (1974). Defence in Animals: A Survey of Anti‐predator Defences. Longmans.

[ece37213-bib-0019] Emberts, Z. , Escalante, I. , & Bateman, P. W. (2019). The ecology and evolution of autotomy. Biological Reviews, 94(6), 1881–1896. 10.1111/brv.12539 31240822

[ece37213-bib-0020] Fleming, P. A. , Valentine, L. E. , & Bateman, P. W. (2013). Telling tails: Selective pressures acting on investment in lizard tails. Physiological and Biochemical Zoology, 86(6), 645–658. 10.1086/673864 24241062

[ece37213-bib-0021] Fresnillo, B. , Belliure, J. , & Cuervo, J. J. (2015). Red tails are effective decoys for avian predators. Evolutionary Ecology, 29(1), 123–135. 10.1007/s10682-014-9739-2

[ece37213-bib-0022] Hartig, F. (2020). DHARMa: Residual Diagnostics for ## Hierarchical (Multi‐Level / Mixed) Regression Models. R Package ## Version 0.3.3.0.

[ece37213-bib-0023] Hawlena, D. (2006). Blue tail and striped body: Why do lizards change their infant costume when growing up? Behavioral Ecology, 17(6), 889–896. 10.1093/beheco/arl023

[ece37213-bib-0024] Heninger, R. , Watson, C. M. , & Cox, C. L. (2020). Relative fitness of decoy coloration is mediated by habitat type. Zoology, 142, 125820. 10.1016/j.zool.2020.125820 32769003

[ece37213-bib-0025] Higham, T. E. , Lipsett, K. R. , Syme, D. A. , & Russell, A. P. (2013). Controlled chaos: Three‐dimensional kinematics, fiber histochemistry, and muscle contractile dynamics of autotomized lizard tails. Physiological and Biochemical Zoology, 86(6), 611–630. 10.1086/673546 24241060

[ece37213-bib-0026] Higham, T. E. , & Russell, A. P. (2010). Flip, flop and fly: Modulated motor control and highly variable movement patterns of autotomized gecko tails. Biology Letters, 6(1), 70–73. 10.1098/rsbl.2009.0577 19740891 PMC2817253

[ece37213-bib-0027] Humphreys, R. K. , & Ruxton, G. D. (2018). What is known and what is not yet known about deflection of the point of a predator’s attack. Biological Journal of the Linnean Society, 123(3), 483–495. 10.1093/biolinnean/blx164

[ece37213-bib-0028] Kacelnik, A. , & Bateson, M. (1996). Risky theories—the effects of variance on foraging decisions. American Zoologist, 36(4), 402–434. 10.1093/icb/36.4.402

[ece37213-bib-0029] Kacelnik, A. , & El Mouden, C. (2013). Triumphs and trials of the risk paradigm. Animal Behaviour, 86(6), 1117–1129. 10.1016/j.anbehav.2013.09.034

[ece37213-bib-0030] Kjernsmo, K. , & Merilaita, S. (2013). Eyespots divert attacks by fish. Proceedings of the Royal Society: B, 280(1766), 20131458. 10.1098/rspb.2013.1458 23864602 PMC3730605

[ece37213-bib-0031] Maginnis, T. L. (2006). The costs of autotomy and regeneration in animals: A review and framework for future research. Behavioral Ecology, 17(5), 857–872. 10.1093/beheco/arl010

[ece37213-bib-0032] Mowat, F. M. , Petersen‐Jones, S. M. , Williamson, H. , Williams, D. L. , Luthert, P. J. , Ali, R. R. , & Bainbridge, J. W. (2008). Topographical characterization of cone photoreceptors and the area centralis of the canine retina. Molecular Vision, 14, 2518. 19112529 PMC2610288

[ece37213-bib-0033] Murali, G. , & Kodandaramaiah, U. (2016). Deceived by stripes: Conspicuous patterning on vital anterior body parts can redirect predatory strikes to expendable posterior organs. Royal Society Open Science, 3(6), 160057. 10.1098/rsos.160057 27429765 PMC4929900

[ece37213-bib-0034] Murali, G. , & Kodandaramaiah, U. (2018). Body size and evolution of motion dazzle coloration in lizards. Behavioral Ecology, 29(1), 79–86. 10.1093/beheco/arx128

[ece37213-bib-0035] Murali, G. , Merilaita, S. , & Kodandaramaiah, U. (2018). Grab my tail: Evolution of dazzle stripes and colourful tails in lizards. Journal of Evolutionary Biology, 31(11), 1675–1688. 10.1111/jeb.13364 30102810

[ece37213-bib-0036] Naya, D. E. , Veloso, C. , Muñoz, J. L. P. , & Bozinovic, F. (2007). Some vaguely explored (but not trivial) costs of tail autotomy in lizards. Comparative Biochemistry and Physiology Part A: Molecular & Integrative Physiology, 146(2), 189–193. 10.1016/j.cbpa.2006.10.014 17113802

[ece37213-bib-0037] Neitz, J. , Geist, T. , & Jacobs, G. H. (1989). Color vision in the dog. Visual Neuroscience, 3(2), 119–125. 10.1017/S0952523800004430 2487095

[ece37213-bib-0038] Ortega, J. , López, P. , & Martín, J. (2014). Conspicuous blue tails, dorsal pattern morphs and escape behaviour in hatchling Iberian wall lizards (*Podarcis hispanicus*). Biological Journal of the Linnean Society, 113(4), 1094–1106. 10.1111/bij.12379

[ece37213-bib-0039] Pafilis, P. , Foufopoulos, J. , Poulakakis, N. , Lymberakis, P. , & Valakos, E. D. (2009). Tail shedding in island lizards [Lacertiidae, Reptilia]: Decline of anitpredator defences in relaxed predation environments. Evolution, 63(5), 1262–1278. 10.1111/j.1558-5646.2009.00635.x 19187256

[ece37213-bib-0040] Pérez‐Mellado, V. , Corti, C. , & Lo Cascio, P. (1997). Tail autotomy and extinction in Mediterranean lizards. A preliminary study of continental and insular populations. Journal of Zoology, 243(3), 533–541. 10.1111/j.1469-7998.1997.tb02799.x

[ece37213-bib-0041] Powell, R. A. (1982). Evolution of black‐tipped tails in weasels: Predator confusion. The American Naturalist, 119(1), 126–131. 10.1086/283897

[ece37213-bib-0042] R Core Team (2018). R: A language and environment for statistical computing. In R Foundation for Statistical Computing (3.5). http://www.r‐project.org

[ece37213-bib-0043] Ruxton, G. D. , Allen, W. L. , & Sherratt, T. N. (2018). Avoiding Attack: The Evolutionary Ecology of Crypsis, Mimicry and Warning Signals, 2nd ed. Oxford University Press. 10.1093/oso/9780199688678.001.0001

[ece37213-bib-0044] Schaefer, H. M. , & Ruxton, G. D. (2009). Deception in plants: Mimicry or perceptual exploitation? Trends in Ecology & Evolution, 24(12), 676–685. 10.1016/j.tree.2009.06.006 19683828

[ece37213-bib-0045] Schneider, C. A. , Rasband, W. S. , & Eliceiri, K. W. (2012). NIH Image to ImageJ: 25 years of image analysis. Nature Methods, 9(7), 671–675. 10.1038/nmeth.2089 22930834 PMC5554542

[ece37213-bib-0046] Siddiqi, A. (2004). Interspecific and intraspecific views of color signals in the strawberry poison frog *Dendrobates pumilio* . Journal of Experimental Biology, 207(14), 2471–2485. 10.1242/jeb.01047 15184519

[ece37213-bib-0047] Stephens, D. W. (1981). The logic of risk‐sensitive foraging preferences. Animal Behaviour, 29(2), 628–629. 10.1016/S0003-3472(81)80128-5

[ece37213-bib-0048] Telemeco, R. S. , Baird, T. A. , & Shine, R. (2011). Tail waving in a lizard (*Bassiana duperreyi*) functions to deflect attacks rather than as a pursuit‐deterrent signal. Animal Behaviour, 82(2), 369–375. 10.1016/j.anbehav.2011.05.014

[ece37213-bib-0049] Troscianko, J. , & Stevens, M. (2015). Image calibration and analysis toolbox – a free software suite for objectively measuring reflectance, colour and pattern. Methods in Ecology and Evolution, 6(11), 1320–1331. 10.1111/2041-210X.12439 27076902 PMC4791150

[ece37213-bib-0050] Van Buskirk, J. , Aschwanden, J. , Buckelmüller, I. , Reolon, S. , & Rüttiman, S. (2004). Bold tail coloration protects tadpoles from dragonfly strikes. Copeia, 2004(3), 599–602. 10.1643/CE-03-283R

[ece37213-bib-0051] Vorobyev, M. , & Osorio, D. (1998). Receptor noise as a determinant of colour thresholds. Proceedings of the Royal Society of London. Series B: Biological Sciences, 265(1394), 351–358. 10.1098/rspb.1998.0302 9523436 PMC1688899

[ece37213-bib-0052] Watson, C. M. , Roelke, C. E. , Pasichnyk, P. N. , & Cox, C. L. (2012). The fitness consequences of the autotomous blue tail in lizards: An empirical test of predator response using clay models. Zoology, 115(5), 339–344. 10.1016/j.zool.2012.04.001 22938695

